# Morphological and Physiological Characteristics of Ruptured Plaques in Native Arteries and Neoatherosclerotic Segments: An OCT-Based and Computational Fluid Dynamics Study

**DOI:** 10.3389/fcvm.2022.890799

**Published:** 2022-05-26

**Authors:** Chongying Jin, Ryo Torii, Anantharaman Ramasamy, Vincenzo Tufaro, Callum D. Little, Klio Konstantinou, Yi Ying Tan, Nathan A. L. Yap, Jackie Cooper, Tom Crake, Constantinos O’Mahony, Roby Rakhit, Mohaned Egred, Javed Ahmed, Grigoris Karamasis, Lorenz Räber, Andreas Baumbach, Anthony Mathur, Christos V. Bourantas

**Affiliations:** ^1^Department of Cardiology, Barts Heart Centre, Barts Health NHS Trust, London, United Kingdom; ^2^Department of Cardiology, Sir Run Run Shaw Hospital, School of Medicine, Zhejiang University, Hangzhou, China; ^3^Centre for Cardiovascular Medicine and Devices, William Harvey Research Institute, Queen Mary University of London, London, United Kingdom; ^4^Department of Mechanical Engineering, University College London, London, United Kingdom; ^5^Department of Biomedical Sciences, Humanitas University, Milan, Italy; ^6^Royal Free Hospital, University College London, London, United Kingdom; ^7^Essex Cardiothoracic Centre, Anglia Ruskin School of Medicine, Essex, United Kingdom; ^8^Barts and The London School of Medicine and Dentistry, London, United Kingdom; ^9^Institute of Cardiovascular Science, University College London, London, United Kingdom; ^10^Freeman Hospital, Newcastle University, Newcastle upon Tyne, United Kingdom; ^11^Department of Cardiology, University of Bern, Bern, Switzerland; ^12^Yale University School of Medicine, New Haven, CT, United States

**Keywords:** plaque rupture, neoatherosclerosis, optical coherence tomography, computational fluid dynamics (CFD), endothelial shear stress, plaque structural stress

## Abstract

**Background:**

Intravascular imaging has been used to assess the morphology of lesions causing an acute coronary syndrome (ACS) in native vessels (NV) and identify differences between plaques that ruptured (PR) and caused an event and those that ruptured without clinical manifestations. However, there is no data about the morphological and physiological characteristics of neoatherosclerotic plaques that ruptured (PR-NA) which constitute a common cause of stent failure.

**Methods:**

We retrospectively analyzed data from patients admitted with an acute myocardial infarction that had optical coherence tomography (OCT) imaging of the culprit vessel before balloon pre-dilation. OCT pullbacks showing PR were segmented at every 0.4 mm. The extent of the formed cavity, lipid and calcific tissue, thrombus, and macrophages were measured, and the fibrous cap thickness (FCT) and the incidence of micro-channels and cholesterol crystals were reported. These data were used to reconstruct a representative model of the native and neoatherosclerotic lesion geometry that was processed with computational fluid dynamics (CFD) techniques to estimate the distribution of the endothelial shear stress and plaque structural stress.

**Result:**

Eighty patients were included in the present analysis: 56 had PR in NV (PR-NV group) and 24 in NA segments (PR-NA group). The PR-NV group had a larger minimum lumen area (2.93 ± 2.03 vs. 2.00 ± 1.26 mm^2^, *p* = 0.015) but similar lesion length and area stenosis compared to PR-NA group. The mean FCT (186 ± 65 vs. 232 ± 80 μm, *p* = 0.009) and the lipid index was smaller (16.7 ± 13.8 vs. 25.9 ± 14.1, *p* = 0.008) while the of calcific index (8.3 ± 9.5 vs. 2.2 ± 1.6%, *p* = 0.002) and the incidence of micro-channels (41.4 vs. 12.5%, *p* = 0.013) was higher in the PR-NV group. Conversely, there was no difference in the incidence of cholesterol crystals, thrombus burden or the location of the rupture site between groups. CFD analysis revealed higher maximum endothelial shear stress (19.1 vs. 11.0 Pa) and lower maximum plaque structural stress (38.8 vs. 95.1 kPa) in the PR-NA compared to the PR-NV model.

**Conclusion:**

We reported significant morphological and physiological differences between culprit ruptured plaques in native and stented segments. Further research is needed to better understand the causes of these differences and the mechanisms regulating neoatherosclerotic lesion destabilization.

## Introduction

Ischemic heart disease (IHD) is the leading cause of death in the world, associated with increased morbidity and devastating financial consequences. Percutaneous coronary interventions (PCI) is an established therapy for patients with IHD and has been associated with better prognosis in acute coronary syndromes (ACS) and improved quality of life in chronic coronary syndromes (1–3). Despite the advances in stent technology that have improved stent safety profile and efficacy enabling treatment of high-risk patients and complex lesions, this treatment can fail and it appears unable to inhibit atherosclerotic disease progression in the treated segments. Numerous intravascular imaging studies have shown that neointima tissue can develop within stented segments and evolve to neoatherosclerotic high-risk vulnerable plaques which can rupture and cause adverse events (4, 5). Several reports attempted to identify predictors of neoatherosclerosis showing that conventional risk factors associated with atherosclerotic evolution, such as baseline demographics (i.e., renal failure, hypercholesterolemia, and hypertension), medications and stent type determine the long-term vessel response to therapy and are associated with the formation of intra-stent high-risk lesions (6–8). In addition, there is an association between development of neoatherosclerotic plaques and atherosclerotic disease progression in native vessels (NV) suggesting these two pathologies are regulated by similar pathophysiological mechanisms (9).

Apart from systemic factors, focal pathobiological mechanisms that are not seen in NV – such as hypersensitivity reaction to stent polymer or endothelial dysfunction induced by eluted drugs – are also involved in neoatherosclerotic lesion formation (4). The different pathogenesis of these lesions is likely to have an impact not only on their development and distribution in stented segments, but also on their destabilization and rupture (10). The objectives of the present analysis are to compare the phenotypic characteristics of lesions that ruptured and caused events in native and stented segments, reconstruct a representative geometry of a ruptured plaque in a native and stented segment and process these models with computational fluid dynamic (CFD) techniques to assess the effect of lesions physiology [endothelial shear stress (ESS) and plaque stress] on their destabilization.

## Materials and Methods

### Study Population

We retrospectively enrolled patients with ST-elevation or non-ST-elevation myocardial infarction who had optical coherence tomography (OCT) of the culprit vessel prior to balloon predilatation. These patients were admitted between 2016 and 2019 in six hospitals: Barts Health NHS Trust, United Kingdom; Bern University Hospital, Switzerland; Royal Free Hospital, United Kingdom; Newcastle Upon Tyne NHS Foundation Trust, United Kingdom; Essex Cardiothoracic Centre, United Kingdom; and Sir Run Run Shaw Hospital, China.

### Optical Coherence Tomography Image Acquisition

OCT imaging was performed using either C7XR, OPTIS™ (St-Jude Medical, Westford, MA, United States), or Lunawave (Terumo Corp., Tokyo, Japan) Fourier Domain system. Pullbacks were performed by using either a manual or automatic blood flushing at a constant speed ranging from 18–40 mm/s and frame rate that ranged from 100 to 180 fr/s. The collected data were stored in DICOM format and sent to Barts Heart Centre for analysis.

### Optical Coherence Tomography Data Analysis

The anonymized data were reviewed by two experienced analysts (CJ and CB) and the culprit lesions were identified. Plaque rupture was defined as the presence of a fibrous cap discontinuity which connected the lumen and the inner – the necrotic core – of the plaque (11). Depending on culprit lesion location these were divided in two groups: plaque ruptures occurring in a native vessel (PR-NV) and those occurring in a neoatherosclerotic lesion (PR-NA). Cases where the plaque rupture extending across the stent edge to the native artery segment were excluded. Moreover, we excluded cases of neoatherosclerotic plaque rupture within a previously implanted bioresorbable scaffold as these devices tend to resorb with time and thus alter ESS and plaque structural stress (PSS) distribution.

The two expert analysts who had established reproducibility and were blinded to patients’ characteristics performed imaging analysis using the QCM-CMS version 4.69 software (Leiden University Medical Center, Leiden, Netherlands) (10, 12). In the PR-NV group, the ruptured plaque was detected and its proximal and distal end was defined as the last and first frame that portrayed lipid tissue or had a plaque burden ≥40% (13). In the PR-NA group, the proximal and distal end of the ruptured plaque was defined as the most proximal and distal frame that had a mean neointima thickness ≥0.5 mm; in case of lesions extending beyond the stented segment, the same definition was used for the PR-NV group to define its proximal or distal end (14).

**FIGURE 1:**
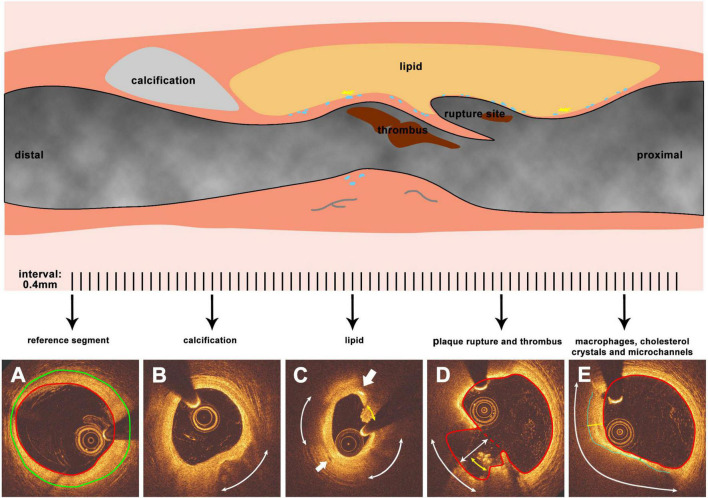
Representative images and methodology of OCT morphology analysis. The upper part is a representative longitudinal diagram of a ruptured plaque (blue dots represent for macrophages while yellow sharp stars for cholesterol crystals, gray curved lines for microchannels), panels **(A–E)** are cross sections corresponding to the lesion segment while analyzed at 0.4 mm interval. **(A)** The reference segment of the vessel, red line indicates the lumen surface while green line indicates the EEM. **(B)** Calcification tissue in the plaque, the arc indicates the calcific arc. **(C)** Lipid tissue in the plaque, the arc indicates the lipid arc, the area enclosed by the blue line indicates the fibrous cap. Yellow and green lines which are vertical to the lumen border are maximum and minimal cap thickness respectively. **(D)** Plaque rupture and thrombus. Red line represents the lumen surface while red dash line indicates the lumen surface before plaque rupture. The white arc represent rupture circumferential extend, while yellow arc indicates thrombus circumferential distribution for generating the thrombus score. Line with arrowheads represents the depth of rupture. **(E)** Macrophages, cholesterol crystals, and microchannels. The bigger arrow points to the cholesterol crystals while the smaller arrow points to the microchannel. Arcs indicate the arc of macrophages.

OCT pullbacks were analyzed at 0.4 mm interval (0.375 mm for Lunawave system). In the PR-NV group, the external elastic membrane (EEM) was detected, when it was visible, and used to define the proximal and distal end of the culprit lesion, while in the PR-NA group the lumen and the stent borders were identified, the mean neointima thickness was measured and the most proximal and distal frames of the lesion with thickness ≥0.5 mm defined its proximal and distal end. Then, the plaque composition was estimated using established criteria. A lipid pool was defined as a low signal intensity region with a circumferential arc >45° with diffuse borders and increased attenuation, while calcification was characterized as a low backscattering region with clear borders (13, 15). The lateral borders of these were detected, and the lipid and calcific arc were estimated. The fibrous cap over lipid tissue was extracted using semi-automated methodology and the minimum and mean cap thickness were computed (16). Macrophages infiltration was defined as punctuated or signal-rich regions with strong signal attenuation behind. Macrophages may appear as dots (single bright spots or cluster of dots) or lines (confluent accumulations forming a thin bright line) with lateral extent >20° (13). Micro-channels were characterized as continuous signal poor holes in three consecutive frames with a diameter ≤300 μm that were not connected to the lumen (15). Cholesterol crystals were defined as sharp, linear regions with high signal intensity (15, 17). Thrombus was classified as a mass in the lumen or attached to the lumen surface with a dimension ≥250 μm (Figure 1) (18).

A lesion was defined as fibroatheroma when it contained more than a quadrant of lipid tissue; the minimum cap thickness over this tissue type was used to classify lesions as thin (TCFA, minimum cap thickness: ≤65 μm) or thick cap fibroatheroma (ThCFA, minimum cap thickness: >65 μm) (11). The length, the maximum and mean arc of lipid tissue, calcification and lined macrophages were measured, and the lipid/calcification/macrophages index were calculated and defined as: 100 × (mean arc in degrees × length) / (lesion length × 360°) (19). In frames portraying a ruptured plaque, an additional border was drawn that connected the edges of the ruptured fibrous cap and represented the lumen surface before plaque/neointima rupture (20). This was used to define the maximum circumferential extend and depth of rupture (defined as the maximum distance between the approximated lumen and the lumen border of the cavity); in addition, the rupture extent index was defined as: 100 × (mean rupture arc × rupture length) / (lesion length × 360°) (19). The thrombus score was computed for each lesion as the sum of the quadrants with thrombus in the analyzed cross-section images divided by the total number of quadrants (21).

**FIGURE 2:**
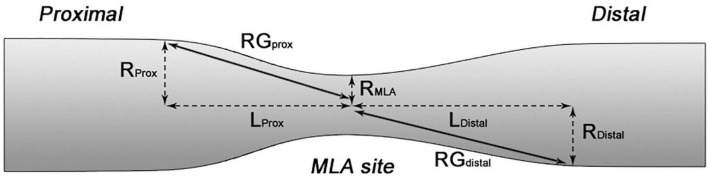
Schematic diagram showing the calculation of RG RG_*prox*_ = (R_*Prox*_-R_*MLA*_)/L_*Prox*_ while RG_*distal*_ = (R_*Distal*_-R_*MLA*_)/L_*Distal*_.

Each lesion was split in three segments: the upstream – defined as the segment between the proximal end of the lesion and 2.5 mm proximal to the minimum lumen area (MLA) – the MLA site – defined as the segment 2.5 mm proximally and distally to the MLA – and the downstream – defined as the segment between 2.5 mm distally to the MLA and the distal end of the lesion. For the upstream and downstream segments, we estimated the radius gradient (RG) which provides an assessment of lesion longitudinal geometry and has been found to be predictor of the location of the ruptured site; this was calculated as: (lumen radius at the proximal or distal end of the ruptured plaque – radius at the MLA site)/the length of the upstream or downstream segment respectively (Figure 2) (22).

### Generation of Mean Anatomical Model

The mean geometrical characteristics of ruptured plaque were used to generate a representative idealized geometry of the culprit lesion before its rupture for PR-NV and PR-NA groups. For this purpose, the lumen surface before plaque/neointima rupture was used to estimate the mean proximal and distal reference lumen diameter and the mean minimum lumen diameter. In addition, the mean lesion length, the minimum and mean fibrous cap thickness (FCT), the mean lipid index and the mean location of the lipid tissue with regards to the MLA were estimated in the two groups. The stent was assumed to have a typical geometry of a drug eluting stent (Endeavor Zotarolimus Eluting Stent, Medtronic, Santa Rosa, CA, United States) with circular struts with a thickness of 90 μm and inter-strut distance of 1 mm. In the PR-NA the mean stent diameter was computed from the struts in OCT images while in the PR-NV group the EEM that was visible at the proximal and distal end of the lesions was used to estimate the mean EEM at the reference sites. At the MLA site where the EEM was not visible this was estimated assuming a remodeling index of 1.20 based on published literature assessing ruptured plaque characteristics using intravascular ultrasound imaging (21, 23). The above values were used to reconstruct pre-rupture plaque geometries in the PR-NV and PR-NA groups assuming that the vessels were straight.

### Computational Fluid and Solid Mechanics Analysis

The two models were meshed with tetrahedral and prismatic cells for CFD analyses using ANSYS Workbench (version 2019 R3, ANSYS Inc., Cannonsburg, MI, United States). For the wall domain, the model was cut in half in reference to the symmetry plane, in order to reduce the computational cost. The resolution of computational meshes were determined through mesh convergence test, and the final model had 114,150 elements in the lumen and 5,563,033 elements in the wall.

The lumen model was used to compute the ESS by solving the 3D incompressible Navier-Stokes equations using ANSYS CFX. The blood was assumed to be homogeneous and Newtonian fluid with dynamic viscosity of 0.0035 Pa s and a density of 1,050 kg/m^3^. Blood flow was considered to be incompressible and laminar, and steady state condition was assumed. A parabolic velocity profile was specified at the inlet, with 1 ml/s normal coronary inflow for both PR-NV and PR-NV cases (24). At the outlet of each model, a pressure of 100 mmHg representing normal coronary blood pressure was applied. The vessel wall of the two models was considered to be rigid and no-slip conditions were applied at the luminal surface.

The PSS in the neointima or in the plaque was computed using ANSYS Mechanical which solves force equilibrium equations. It was assumed that the pressure on the lumen-wall interface was originated from both static blood pressure and flow stream. The proximal and distal end of the PR-NV and PR-NA models were fixed in space. A 5-parameter Mooney Rivlin non-linear hyperelastic material model (25) was used for the lipid and fibrous components of neointima and the native vessel wall, while stent struts were modeled as a linear elastic material with elastic modulus 243 GPa and Poisson’s ratio 0.29 assuming the material is L605 Co-Cr alloy (26). All computational analyses were conducted on a desktop workstation (Dell Precision 5120, 3.7 GHz Intel Core i9, 128 GB RAM).

### Statistical Analysis

Continuous data are presented as mean ± SD if normally distributed, otherwise as median and interquartile range (IQR). Categorical data are expressed as absolute values and percentages. *T*-test was used to compare continues variables if these were normally distributed and Mann–Whitney U-test when variables were not normally distributed; comparison of categorical variables were performed using χ^2^ test. Analysis was performed using MedCalc Statistical Software version 18.2.1 (MedCalc Software bvba, Ostend, Belgium). A *p*-value of <0.05 was assumed to be statistically significantly.

## Results

### Clinical Data of Study Population

**FIGURE 3:**
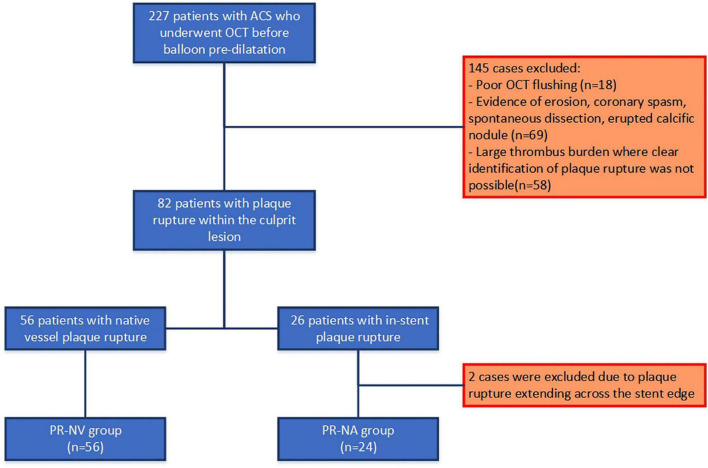
Study flowchart.

Two hundred twenty-seven patients presented with an acute myocardial infarction who underwent OCT before culprit lesion pre-dilatation were considered eligible for recruitment. From these, we excluded patients with non-interpretable OCT due to suboptimal image quality or large thrombus burden, those who suffered an event because of plaque erosion, coronary spasm, spontaneous coronary dissection, or an erupted calcific nodule and two cases because the ruptured lesion was located at the edge of a stent and extended to the native vessel (Figure 3).

Therefore, 80 patients were included in the final analysis: 56 had a ruptured plaque in NV and 24 in NA segment. There was no significant difference in baseline demographics between the two groups except the medication therapy at the event including anti-platelet therapy (defined as continuously taken of either aspirin or P2Y12 inhibitor or both at the event) and statin usage (Table 1). Thrombus aspiration was used in 23 cases (41.1%) in the PR-NV group and 11 patients (45.8%) in the PR-NA group (*p* = 0.693), while glycoprotein IIb/IIIa inhibitors were used during PCI in 14 (25.0%) and 12 patients (50.0%), respectively (*p* = 0.029).

**TABLE 1:** Baseline demographics of the studied population.

	PR-NV group (*n* = 56)	PR-NA group (*n* = 24)	*p*-Value
Mean age (years)	64.5 ± 13.1	66.7 ± 12.8	0.911
Males	45 (80.4)	19 (79.2)	0.904
**Clinical characteristics**			
Diabetes	16 (28.6)	6 (25.0)	0.745
Hypertension	29 (51.8)	14 (58.3)	0.593
Hyperlipidemia	30 (53.6)	17 (70.8)	0.153
Current smoking	19 (33.9)	5 (20.8)	0.245
Previous MI	10 (17.9)	9 (37.5)	0.060
**Clinical presentation**			0.117
STEMI	29 (51.8)	17 (70.8)	
NSTEMI	27 (48.2)	7 (29.2)	
**Medications at the time of the event**			
Anti-platelet therapy	26 (46.4)	18 (75.0)	**0.019**
Aspirin	21 (37.5)	17 (70.8)	**0.006**
P2Y12 inhibitor	5 (8.9)	5 (20.8)	0.157
Statins	27 (48.2)	19 (79.2)	**0.010**
ACEI/ARB	24 (42.9)	15 (62.5)	0.144
β-Blockers	19 (33.9)	14 (58.3)	0.051
**Culprit vessel**			0.896
LAD	29 (51.8)	14 (58.3)	
LCx	12 (21.4)	3 (12.5)	
RCA	15 (26.8)	7 (29.2)	

*Values are presented as n (%) or mean ± SD. MI, myocardial infarction; STEMI, ST-segment elevation myocardial infarction; NSTEMI, non-ST-segment elevation myocardial infarction; ACEI, angiotensin converting enzyme inhibitors; ARB, angiotensin receptor blocker; LAD, left anterior descending; LCx, left circumflex artery; RCA, right coronary artery. The values in bold type indicate that they are statistically significantly with p-value of <0.05.*

### OCT Analysis

#### Lesion Level-Analysis

**FIGURE 4:**
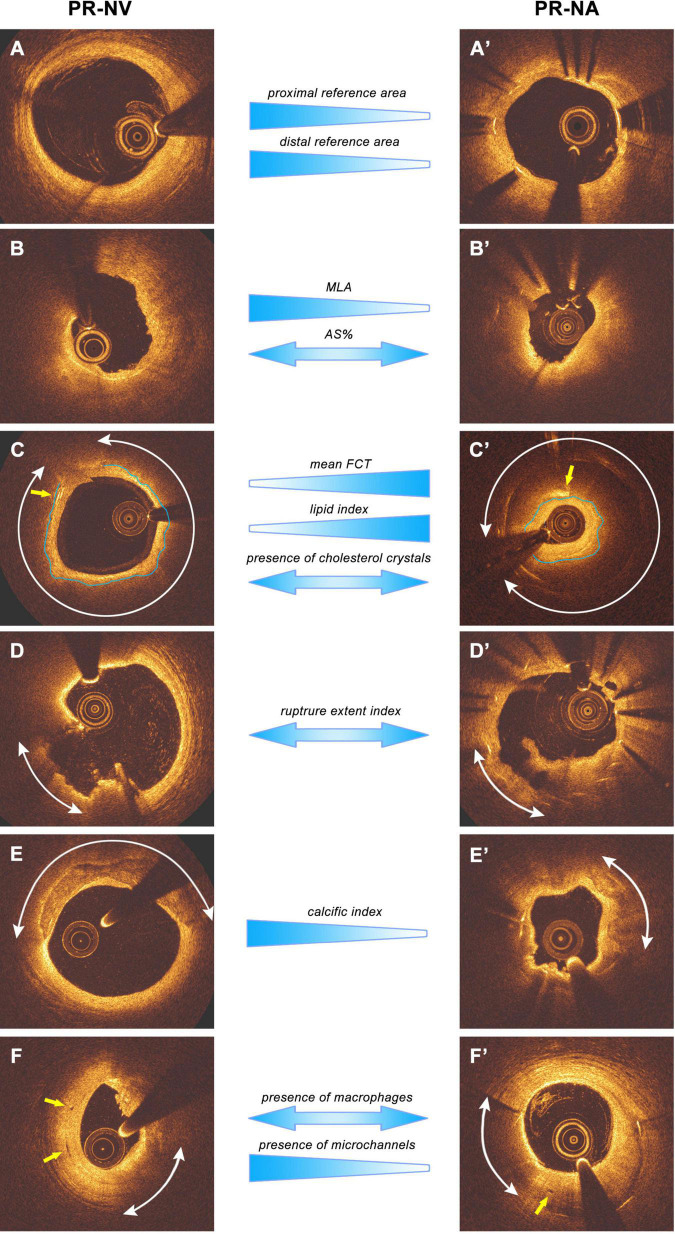
Main results of lesion level-analysis and representative images. The images are presented as pairs from PR-NV and PR-NA groups. **(A,A′)** The reference segment, PR-NV group had larger proximal and distal reference area. **(B,B′)** The MLA site, PR-NV group reveals larger MLA than PR-NA group, while the two groups have similar AS%. **(C,C′)** Typical fibroatheroma and cholesterol crystals images, arcs indicate the lipid tissue while the yellow arrows are pointing at the cholesterol crystals. Blue lines draw the fibrous cap. PR-NV group reveals smaller mean FCT and lipid index, but similar incidence of cholesterol crystals compares to PR-NA group. **(D,D′)** Representative plaque rupture images, arcs indicate the ruptured cavity, PR-NV group reveals similar rupture extend index with PR-NA group. **(E,E′)** Typical calcification images. Arcs indicate the calcific tissue, PR-NV groups had bigger calcific index than PR-NA group. **(F,F′)** Macrophages and microchannels. Yellow arrows are pointing to the microchannels while arcs indicate the macrophages. PR-NV group has similar incidence of macrophages but more incidence of microchannels than PR-NA group.

There was no significant difference in lesion length, area stenosis, phenotype, and number of ruptured sites in the two groups. However, PR-NV lesions had a larger MLA, lager reference areas and smaller mean FCT and small lipid index compared to PR-NA lesions (Table 2). Moreover, the calcific index and the incidence of calcium and microchannels was higher in the PR-NV than the PR-NA group. Conversely, the incidence of macrophages, cholesterol crystals and thrombus were similar in the two groups. Likewise, there were no differences between groups in the length of plaque rupture, the mean arc of rupture, or the rupture extent index as well as the up- and downstream RG, whereas the maximum depth of rupture was greater in the PR-NV group (Figure 4).

**TABLE 2:** Geometrical and morphological characteristics of the ruptured plaques in the native and stented segments.

	PR-NV group (*n* = 56)	PR-NA group (*n* = 24)	*p*-Value
**Lesion-level analysis**			
Geometrical characteristics			
Lesion length (mm)	16.3 ± 6.0	14.8 ± 11.1	0.532
MLA (mm^2^)	2.93 ± 2.03	2.00 ± 1.26	**0.015**
Proximal reference area (mm^2^)	9.21 ± 4.11	5.80 ± 2.99	**<0.001**
Distal reference area (mm^2^)	7.78 ± 3.87	4.96 ± 2.11	**0.001**
Area stenosis (%)	66.4 ± 15.4	61.6 ± 18.1	0.227
Upstream RG	0.111 ± 0.069	0.105 ± 0.076	0.768
Downstream RG	0.112 ± 0.093	0.121 ± 0.117	0.715
**Morphological characteristics**			
TCFA phenotype (*n*, %)	41 (73.2)	14 (58.3)	0.191
ThCFA phenotype (*n*, %)	13 (23.2)	9 (37.5)	0.406
Minimal FCT (μm)	49 ± 22	60 ± 38	0.222
Mean FCT (μm)	186 ± 65	232 ± 80	**0.009**
Mean lipid arc (°)	150.5 ± 49.4	167.3 ± 34.0	0.141
Lipid length (mm)	6.1 ± 5.3	7.8 ± 8.6	0.280
Lipid index	62.2 ± 49.1	97.4 ± 47.8	0.005
Presence of calcific tissue (*n*, %)	31 (55.4)	5 (20.8)	**0.005**
Mean arc of calcific tissue (°)	62.8 ± 31.0	79.2 ± 14.7	0.309
Calcific index	29.9 ± 34.1	10.0 ± 4.0	**0.004**
Presence of macrophages (*n*, %)	52 (92.9)	21 (87.5)	0.440
Spotted macrophages (*n*, %)	50 (89.3)	20 (83.3)	0.464
Lined macrophages (*n*, %)	48 (85.7)	18 (75.0)	0.251
Lined macrophages index	12.1 ± 11.1	26.6 ± 33.9	0.091
Presence of cholesterol crystals (*n*, %)	19 (34.5)	8 (33.0)	0.917
Presence of microchannels (*n*, %)	23 (41.4)	3 (12.5)	**0.013**
Number of rupture sites	1.1 ± 0.4	1.2 ± 0.4	0.473
Length of plaque rupture (mm)	2.4 ± 2.2	1.9 ± 2.0	0.359
Depth of plaque rupture (mm)	0.9 ± 0.5	0.6 ± 0.3	**0.036**
Arc of rupture (°)	58.7 ± 35.1	64.7 ± 31.5	0.476
Rupture extent index	0.8 ± 1.2	0.7 ± 0.6	0.544
Presence of thrombus (*n*, %)	43 (76.8)	21 (87.5)	0.275
Thrombus score	21.8 ± 18.3	21.5 ± 17.3	0.948
**Frame-level analysis**	*n* = 2,307	*N* = 903	
Frames portraying TCFA (*n*, %)	391 (16.9)	121 (13.4)	**0.014**
Frames portraying ThCFA (*n*, %)	458(19.9)	344 (38.1)	**<0.001**
Frames portraying calcific tissue (*n*, %)	433 (18.8)	20 (2.2)	**<0.001**
Frames portraying macrophages (*n*, %)	735 (31.9)	222 (24.6)	**<0.001**
Frames portraying spotted macrophages (*n*, %)	440 (19.1)	112 (12.4)	**<0.001**
Frames portraying lined macrophages (*n*, %)	411 (17.8)	138 (15.3)	0.087
Frames portraying microchannels (*n*, %)	84 (3.6)	28 (3.1)	0.453
Frames portraying cholesterol crystals (*n*, %)	40 (1.7)	30 (3.3)	**0.006**
Frames portraying plaque rupture (*n*, %)	334 (14.5)	114 (12.6)	0.173
Frames portraying thrombus (*n*, %)	576 (25.0)	232 (25.7)	0.671

*Results are presented at a lesion- and frame-level. MLA, minimum lumen area; RG, radius gradient; TCFA, thin cap fibroatheroma; ThCFA, thick cap fibroatheroma; FCT, fibrous cap thickness. The values in bold type indicate that they are statistically significantly with p-value of <0.05.*

#### Frame-Level Analysis

Frame-level analysis showed that PR-NV lesions had more frames with a lipid-rich plaque covered by a thin fibrous cap and more frames covered by a thick cap and portrayed more often calcific tissue, spotted macrophages and less often cholesterol crystals than PR-NA lesions (Table 2).

### Plaque Rupture Location Analysis

Most of plaque ruptures occurred at the throat followed by the upstream region in both native and neoatherosclerotic lesions (for the longitudinal distribution of plaque rupture *p* = 0.098). The distance plaque rupture to MLA was similar in the two groups (Table 3). In addition, there was no differences between groups in the circumferential location of plaque rupture (center vs. shoulder of the plaque, 33 vs. 31 in NV group, and 12 vs. 17 in NA group, *p* = 0.363)

**TABLE 3:** Location of plaque rupture.

	PR-NV group (*n* = 56)	PR-NA group (*n* = 24)	*p*-Value
Distance to MLA site (mm)	3.6 ± 3.9	2.6 ± 3.6	0.679
Upstream (*n*, %)	26 (40.6)	5 (17.2)	
Center (*n*, %)	18 (28.1)	2 (6.9)	
Shoulder (*n*, %)	8 (12.5)	3 (10.3)	
Throat (*n*, %)	30 (46.9)	20 (69.0)	
Center (*n*, %)	12 (18.8)	10 (34.5)	
Shoulder (*n*, %)	18 (28.1)	10 (34.5)	
Downstream (*n*, %)	8 (12.5)	4 (13.8)	
Center (*n*, %)	3 (4.7)	0 (0)	
Shoulder (*n*, %)	5 (7.8)	4 (13.8)	

### Computational Physiological Analysis

**FIGURE 5:**
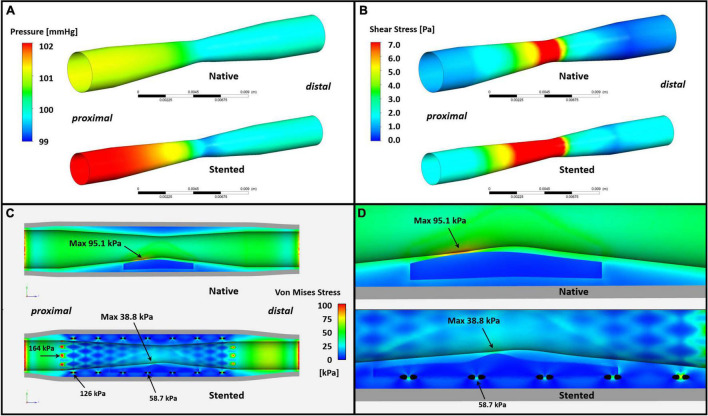
Computational physiological analysis results. **(A)** The pressure drop across the model is 0.95 and 2.03 mmHg for native and stented vessel, respectively. **(B)** ESS distribution of the vessel. The stented model reveals higher maximum and minimum ESS value (19.1 vs. 11.0 Pa and 0.32 vs. 0.04 Pa, respectively). The maximum ESS value was located main at the MLA in both two models and 28.5% of the culprit lesion model in the stented segment and 9.0% of the culprit lesion model in NV was exposed to high ESS (>7 Pa). **(C,D)** PSS analysis results. The maximum superficial PSS in both models were over the thinner segment of the fibrous cap proximally to the MLA and was 95.1 kPa in the native and 38.8 kPa in the stented segment. A total of 95.1 kPa was also the maximum PSS value of the native model while in the stented model the highest PSS was 58.7 kPa in the vicinity of stent struts.

The reconstructed native and stented models are shown in Figure 5. The pressure drop across the native model was 0.95 mmHg, and 2.03 mmHg in the stented model (Figure 5A). The maximum ESS value was higher in the stented model (19.1 vs. 11.0 Pa) and the minimum ESS was lower in the native model (0.32 vs. 0.04 Pa). The maximum ESS value was noted in both models at the MLA; 28.5% of the culprit lesion model in the stented segment and 9.0% of the culprit lesion model in NV was exposed to high ESS (>7 Pa) (27). Furthermore, ESS was higher in the stented model over the minimum FCT which in both models were located proximally to the MLA (Figure 5B).

Plaque structural stress analysis showed significant differences in the two models. The maximum superficial (≤200 μm depth) PSS was noted in both models over the thinner segment of the fibrous cap proximally to the MLA and this was 95.1 kPa in the native and 38.8 kPa in the stented segment. This was also the maximum PSS value in the native culprit plaque; in the stented culprit lesion the highest plaque structural stress was noted in deeper layers in the vicinity of stent struts where its maximum value was 58.7 kPa (Figures 5C,D).

## Discussion

In this study we examined for the first time the morphological and physiological characteristics of lesions located in native and stented segments that ruptured causing a cardiovascular event. We found that (1) there are significant morphological differences between these two groups: ruptured plaques in native segments had larger reference and MLA, an increased calcific tissue component, a thinner fibrous cap, smaller lipid index, and more neo-vessels than the neoatherosclerotic plaques that ruptured and (2) that these morphological differences had an effect of lesion physiology leading to higher ESS and lower superficial PSS in the neoatherosclerotic lesions.

Plaque rupture is considered the main cause of ACS accounting for two thirds of the cardiovascular events (28). Several intravascular imaging studies, over the last years, attempted to assess the morphological characteristics of lesions that ruptured in native segments and caused events showing that these lesions have a small MLA, increased plaque burden and lipid component that is covered by a thin fibrous cap and are often exhibit macrophages accumulations and neovessels (21, 29–31). Conversely, there is lack of evidence about the morphological characteristics of ruptured neoatherosclerotic lesions. In the present analysis we found significant geometrical differences between the lesions in the PR-NV and PR-NA group. Native lesions had a larger MLA and reference lumen areas; this should be attributed that the fact that the vessel wall can remodel and accommodate the developed plaque and to fact that the neoatherosclerotic lesions were developed within stents that were implanted to treat obstructive plaques. Conversely, there were no differences between these groups in lesion length, upstream and downstream RG and the area stenosis. Ruptured neoatherosclerotic lesions similarly to the native lesions were lipid-rich, infiltrated by macrophages and often exhibited cholesterol crystals. On the other hand, the incidence of calcific tissue and micro-channels was lower and the FCT was increased while the lipid index was higher in the PR-NA group; these findings indicate that these lesions ruptured earlier after their development compared to the native that their generation begins in childhood.

Recent data indicate that the pathophysiological pathways that are involved in neoatherosclerotic lesion formation are different to those that regulate plaque evolution in native segments. These include the vessel wall injury post stent implantation, the delayed vascular healing and the endothelial barrier dysfunction caused by the antiproliferative drugs (32, 33), the unfavorable local hemodynamic milieu induced by the protruding struts post stent implantation, that create flow disturbances and recirculation zones in the vicinity of the struts (34), and the vascular inflammation caused by the polymer of the stent (4). The outcome of these processes is the formation of intraplaque hemorrhage derived from the lumen and the accumulation of foamy macrophages in the peri-strut regions that apoptose leading to necrotic core formation (35). In addition, to these mechanisms systemic factors and the patients’ cardiovascular profile and vulnerability seem also to be involved and expedite neoatherosclerotic lesion formation (6–9).

Although several studies have shed light onto the pathophysiological pathways that regulate vulnerable plaque development within the stents, there is limited data about the processes that contribute to their destabilization and rupture. In native lesions, local hemodynamic forces distribution seems to play a pivotal role on the final act of atherosclerosis. More specifically, high ESS promotes nitric oxide synthesis resulting in proteolytic degradation of the fibrous cap, inhibits extracellular matrix synthesis and upregulate smooth muscle cell apoptosis and vascular inflammation leading to fibrous cap thinning and fragility (36, 37). Moreover, PSS has a pivotal role on plaque destabilization as it promotes metalloproteinase synthesis, macrophages accumulation, smooth muscle cell apoptosis, and intraplaque hemorrhage (38–41) and is also considered as the main instigator of plaque rupture (42).

In contrast to the NV in stented segments there is limited evidence about the implications of the local hemodynamic forces on vulnerable plaque formation. Two recent reports have shown that low ESS is a predictor of lipid-rich neointima and neointima inflammation and are associated with the formation of neoatherosclerotic lesions, while high ESS appear to contribute to their destabilization and rupture (14, 43). However, both studies have significant limitations as they included a small number of patients, they made assumptions about vessel geometry post stent implantation, as the baseline intravascular imaging data were not available, and did not assess the PSS and its implication on neoatherosclerotic evolution.

In this analysis we examined for the first time the effect of PSS on neoatherosclerotic plaque rupture. We generated a representative idealized geometry of the culprit lesion before its rupture in native and stented segments and process this with CFD techniques to assess ESS and PSS distribution. We found high ESS values in both geometries with similar ESS distribution and the maximum ESS at the MLA; however, the ESS values were numerically higher in the stented segment. This should be attributed to the smaller MLA in this model. The higher ESS in the neoatherosclerotic lesions is likely to have clinical implications, expedite collagen degradation in the fibrous cap leading to its earlier destabilization compared to the native lesions. This hypothesis may not be supported by the FCT that is numerically higher in the stent geometry; however, it has to be acknowledged that OCT is unable to assess fibrous cap composition and the density of the collagen fibers. Conversely, the superficial PSS was higher in the native model; this should be attributed to the smaller lumen dimensions in the stent model and the fact that the neoatherosclerotic lesions were caged by the deployed stents. Stent struts also affect PSS in the deeper layers of neointima with high PSS values noted at their borders. This may influence neoatherosclerotic lesion formation but also to its destabilization as increased PSS promotes neovessels rupture and intraplaque hemorrhage (40).

### Limitations

Several limitations of the present analysis should be acknowledged. Firstly, the number of the included native and especially of the neoatherosclerotic ruptured plaques is relatively small. This should be attributed to the fact that we included only lesions that were not pre-dilated before OCT imaging and that we excluded 33.5% of the lesions because of insufficient OCT image quality that prohibited image segmentation or because it was not possible to identify the cause the event (plaque rupture vs. plaque erosion vs. calcific nodule). The strict inclusion criteria provided confidence about our findings but may have also introduced bias; nevertheless, it is reassuring that our results in the native lesions are in agreement with those reported in the literature (22). Secondly, the type of stent implanted in the PR-NA group was not always available. Thus it was not possible to assess the effect of different strut configuration polymer and drug elution on the morphological characteristics in the culprit neoatherosclerotic lesions. Thirdly, CFD analysis was performed in a representative idealized model of the stented and native lesions and not in the entire dataset. Therefore, it was not possible to examine the effect of specific morphological characteristics, seen in different lesions, on the distribution of the local hemodynamic forces. This would enable a more accurate assessment of the local hemodynamic milieu and would allow more accurate comparisons between groups. However, this analysis was not feasible as segment reconstruction could not be performed in all frames of the studied lesions, because of the increased thrombus burden and would require increased computational time and resources. Finally, we approximated tissue mechanical properties based on the published literature, and the vessel geometry before rupture as OCT imaging data were not available before the event. Considering however, that prospective large-scale studies that will include asymptomatic patients who will undergo OCT imaging in native and stented segments – so as to capture the morphological features of vulnerable plaques before their rupture – is unlikely to be feasible in future, we believe that the present analysis is important, and it provides valuable mechanistic insights about the role of ESS and PSS on neoatherosclerotic lesion destabilization.

## Conclusion

Neoatherosclerotic lesions that ruptured, and caused events have significant morphological differences from the culprit lesions in native segments. These differences affect local hemodynamic forces distribution resulting in higher ESS and lower superficial PSS in the neoatherosclerotic plaques. These findings highlight the need to conduct large intravascular- and CFD-based studies to better understand the pathophysiological mechanisms that regulate neoatherosclerotic lesion formation and destabilization.

## Data Availability Statement

The original contributions presented in the study are included in the article/supplementary material, further inquiries can be directed to the corresponding author.

## Ethics Statement

Ethical review and approval was not required for this study on human participants in accordance with local legislation and institutional requirements. Written informed consent for participation was not required for this study because the data was anonymized and the study retrospective in nature.

## Author Contributions

CB, AM, LR, and AB contributed to conception and design of the study. CJ, CL, KK, RR, ME, JA, and GK organized the database. CJ, CB, AR, and VT performed OCT analysis. RT and YT performed the CFD analysis. CJ and CB did the statistical analysis. CJ wrote the first draft of the manuscript. AR, VT, NY, TC, JC, and CO’M wrote sections of the manuscript. All authors contributed to manuscript revision, read, and approved the submitted version.

## Conflict of Interest

The authors declare that the research was conducted in the absence of any commercial or financial relationships that could be construed as a potential conflict of interest.

## Publisher’s Note

All claims expressed in this article are solely those of the authors and do not necessarily represent those of their affiliated organizations, or those of the publisher, the editors and the reviewers. Any product that may be evaluated in this article, or claim that may be made by its manufacturer, is not guaranteed or endorsed by the publisher.
